# Supplementary material for: Mechanisms governing the pioneering and redistribution capabilities of the non-classical pioneer PU.1
Source: Nat Commun. 2020 Jan 21;11:402. doi: 10.1038/s41467-019-13960-2 (PMC6972792; doi:10.1038/s41467-019-13960-2)
Supplement: Supplementary file 7 — Source data [file 41467_2019_13960_MOESM7_ESM.zip › Source_Data/FigureS2/FigureS2B_MotifScanOutput/KmeansCluster10/homerResults/motif26.similar.html]

motif26

## Information for motif26

C
T
A
G
A
C
T
G
T
A
C
G
A
T
C
G
A
T
C
G
T
A
C
G
A
C
T
G
  
Reverse Opposite:  

G
T
A
C
A
T
G
C
T
A
G
C
A
T
G
C
A
T
G
C
T
G
A
C
A
G
T
C
  

|  |  |
| --- | --- |
| p-value: | 1e-24 |
| log p-value: | -5.659e+01 |
| Information Content per bp: | 1.766 |
| Number of Target Sequences with motif | 1117.0 |
| Percentage of Target Sequences with motif | 30.09% |
| Number of Background Sequences with motif | 9915.0 |
| Percentage of Background Sequences with motif | 22.44% |
| Average Position of motif in Targets | 100.2 +/- 55.8bp |
| Average Position of motif in Background | 99.0 +/- 64.8bp |
| Strand Bias (log2 ratio + to - strand density) | -0.0 |
| Multiplicity (# of sites on avg that occur together) | 1.30 |
| Motif File: | file (matrix) reverse opposite |

### Similar de novo motifs found

|  |  |  |  |  |  |  |  |
| --- | --- | --- | --- | --- | --- | --- | --- |
| Rank | Match Score | Redundant Motif | P-value | log P-value | % of Targets | % of Background | Motif file |
| 1 | 0.783 | C G A T A C T G A C T G C T A G A C T G A C T G C G T A A C T G A C G T | 1e-18 | -42.215168 | 13.47% | 8.85% | motif file (matrix) |
| 2 | 0.912 | T C A G A T C G C T A G T C A G A T C G C T A G A C T G T A C G | 1e-17 | -40.303042 | 15.46% | 10.62% | motif file (matrix) |
